# Crystal structure of RuvC resolvase in complex with Holliday junction substrate

**DOI:** 10.1093/nar/gkt769

**Published:** 2013-08-24

**Authors:** Karolina M. Górecka, Weronika Komorowska, Marcin Nowotny

**Affiliations:** Laboratory of Protein Structure, International Institute of Molecular and Cell Biology, 4 Trojdena Street, 02-109, Warsaw, Poland

## Abstract

The key intermediate in genetic recombination is the Holliday junction (HJ), a four-way DNA structure. At the end of recombination, HJs are cleaved by specific nucleases called resolvases. In Gram-negative bacteria, this cleavage is performed by RuvC, a dimeric endonuclease that belongs to the retroviral integrase superfamily. Here, we report the first crystal structure of RuvC in complex with a synthetic HJ solved at 3.75 Å resolution. The junction in the complex is in an unfolded 2-fold symmetrical conformation, in which the four arms point toward the vertices of a tetrahedron. The two scissile phosphates are located one nucleotide from the strand exchange point, and RuvC approaches them from the minor groove side. The key protein–DNA contacts observed in the structure were verified using a thiol-based site-specific cross-linking approach. Compared with known complex structures of the phage resolvases endonuclease I and endonuclease VII, the RuvC structure exhibits striking differences in the mode of substrate binding and location of the cleavage site.

## INTRODUCTION

Genetic recombination is used to rearrange genes between homologous sequences and consequently generate genetic diversity and promote evolution. It also serves to preserve genetic information during the repair of double-strand breaks (1). The key intermediates in this process are four-way Holliday junction (HJ) structures (2). They are dynamic and can adopt conformations between two extremes. The first is observed in the absence of metal ions, when repulsion between the negative charges of the DNA backbone forces the four arms to extend toward the corners of a square, thus forming a square planar conformation. In the second conformation, observed when divalent metal ions are present, pairs of the arms stack on each other in an anti-parallel fashion, forming a stacked-X conformation with 2-fold symmetry (3,4).

At the end of the recombination process, HJs must be removed, and one pathway to achieve this is cleavage by specific nucleases called resolvases. Resolvases from different kingdoms of life exhibit remarkable diversity and belong to various classes of nucleases. For example, phage T7 endonuclease I, archeal Hjc and the eukaryotic MUS81-EME1 complex belong to the PD-(D/E)XK nuclease family (5–8). GEN1 in mammals and its ortholog Yen1 in yeast (9) adopt a flap endonuclease fold, and the SLX1-SLX4 complex belongs to the GIY-YIG nuclease class (10).

RuvC resolvase from Gram-negative bacteria belongs to the retroviral integrase superfamily with a characteristic RNase H fold (11). It was the first cellular resolvase to be characterized biochemically (12–14). The RuvC counterparts in yeast are the mitochondrial proteins CCE1 from *Saccharomyces cerevisae* and Ydc2 from *Schizosaccharomyces pombe* (15). In bacteria, RuvC is a part of a system responsible for HJ migration, comprising the HJ-binding protein RuvA and helicase RuvB (1). Electron microscopy data, together with the crystal structures, led to a RuvA/RuvB branch migration complex model (16). In this model, the HJ is sandwiched between two RuvA monomers and two RuvB hexamers positioned perpendicularly to the HJ plane pull on two opposite arms of the junction.

RuvC is a dimeric endonuclease that requires divalent metal ions for activity (17). RuvC resolves HJs by symmetrically cleaving two of its DNA strands (13). Substrate binding does not depend on the DNA sequence, but the cleavage is specific and occurs preferentially at the (A/T)TT↓(G/C) cognate sequence (18,19). The first RuvC protein structure was reported in 1994 (20), revealing the architecture of the dimer with two active sites located 30 Å from each other and formed by four conserved carboxylates (D7, E66, D138 and D141 in *Escherichia coli* protein). These active sites coordinate catalytic metal ions. Recently, also the structures of *Thermus thermophilus* and phage RuvC have been reported (21,22), but to date, the structure in complex with the DNA substrate has remained elusive.

Here, we present the crystal structure of *T. thermophilus* RuvC in complex with a short synthetic HJ solved at 3.75 Å resolution. The structure reveals a RuvC DNA binding interface and a 2-fold symmetrical conformation of the junction. The structure allows us to compare the mode of substrate binding between RuvC and phage resolvases T4 endonuclease VII and T7 endonuclease I, revealing that RuvC exhibits a novel mode of HJ binding.

## MATERIALS AND METHODS

### Protein preparation and solution of the structures

A full description of the methods can be found in the Supplementary Information. Briefly, *T. thermophilus* His-tagged RuvC was expressed in *E. coli* and purified on a nickel column, followed by tag cleavage and further purification on a Mono-S column. Crystals of the apo protein were obtained with 0.1 M citric acid (pH 3.5) and 1.5 M NaCl. The diffraction data from these crystals were collected on a Berliner Elektronenspeicherring-Gesellschaft für Synchrotronstrahlung (BESSY) synchrotron at beamline MX-14.2 on a Mar225 CCD detector at 100 K. The structure was solved using single-wavelength anomalous diffraction (SAD) using data collected from selenomethionine-substituted protein crystals. This structure was used to solve the structure of protein–DNA complex. The crystals of the Tt-RuvC-DNA complex were obtained with oligonucleotides J221 and J222 (Supplementary Table S1) and grown in 0.3–0.5 M ammonium phosphate. Diffraction data sets from these crystals at 3.75 Å resolution were collected at microfocus beamline 23-2 at the European Synchrotron Radiation Facility at 100 K on a Mar225 CCD detector. The structure was solved using molecular replacement (MR) with Phaser (23). The complete model of the complex was built in Coot and refined in phenix.refine (version 1.8.2-1309) (24) with rounds of manual building (Table 1). According to Molprobity analysis, 97.7% of the residues are found in the most favored regions of the Ramachandran plot.

**Table 1.** Data collection and refinement statistics

	apo Tt-RuvC	Tt-RuvC DNA complex
	SAD data	Native data
Space group	*P* 2_1_2_1_2_1_	*P* 3_2_21
Cell dimensions		
*a*, *b*, *c* (Å)	35.3, 61.3,135.6	106.4, 106.4, 132.6
α, β, γ (°)	90, 90, 90	90, 90, 120
Wavelength (Å)	0.97973	0.87260
Resolution (Å)^a^	50-1.35 (1.43–1.35)	50-3.75 (3.96–3.75)
CC ½	99.8 (80.4)	99.7 (77.0)
*I*/σ*I*	13.5 (2.2)	13.2 (2.2)
Completeness (%)	99.7 (99.4)	99.5 (97.7)
	Solutions from SOLVE: Figure-of-merit = 0.48 for 4 Se atoms	
Refinement		
Resolution (Å)	1.35	3.75
No. reflections	655 942	9255
*R*_work_/*R*_free_ (%)	16.0/20.0	26.7/32.9
Solvent content (%)	40.2	68.3
No. atoms	2944 (ASU contains RuvC dimer)	3361 (ASU contains RuvC dimer and a HJ molecule)
Protein	2513	2243
Nucleic acids		1118
Waters/ligands	431	
Avg. *B*-factor	19.8	181.6
Protein	17.0	172.5
Nucleic acids		199.7
Waters/ligands	36.0	
Root-mean-square deviations		
Bond lengths (Å)	0.006	0.0170
Bond angles (°)	1.04	1.952

^a^Values in parentheses are for highest-resolution shell.

SAD, single-wavelength anomalous diffraction.

### Cross-linking experiments

Two cross-linking methods were used: via DNA bases and via DNA backbone. Base-modified oligonucleotides with 2-F-dI in specific positions were obtained from Metabion International AG (Martinsried, Germany) and derivatized with cysteamine. Backbone-modified oligonucleotides were purchased from Future Synthesis (Poznan, Poland). Synthesis of these modified oligonucleodies was based on the H-phosphonate chemistry. Cysteine was condensated with diester H-phosphonate with subsequent oxidation in presence of CCl4. The standard cross-linking reaction mixture (15 µl) contained cysteine-substituted RuvC and the modified substrate in a 1:1 molar ratio. The reaction buffer contained 100 mM NaCl, 10 mM HEPES (pH 7.0), 0.5 mM DTT, 15% glycerol and 5 mM MgCl_2_ for base modifications or the same buffer with the addition of 100 mM KCl for backbone modifications. The samples were incubated for 2.5 h at 37°C, followed by overnight incubation at 25°C. The reaction was stopped by the addition of sample buffer, and the products of the cross-linking reaction were analyzed on precast gradient gels (NuPAGE Bis-Tris Mini Gels 4–12%, Invitrogen) stained with Bio-Safe Coomassie Stain (Bio-Rad). The reaction products were visualized with Image Quant LAS400 scanner (GE Healthcare).

## RESULTS

### Structure determination

To gain insights into substrate recognition by RuvC, we sought to solve its crystal structure in complex with an HJ. We cloned, expressed and purified the RuvC protein from *T. thermophilus* (Tt-RuvC) and verified its enzymatic activity (Supplementary Figure S1). To inhibit HJ cleavage during crystallization, we also prepared an inactive Tt-RuvC variant with E70Q substitution at the active site (Supplementary Figure S1). Tt-RuvC underwent extensive crystallization trials in the presence of short synthetic HJs. Their sequence was based on the published oligonucleotides used for biochemical studies of RuvC (25). The lengths of the arms were chosen based on available footprinting data (26), and shorter arms were stabilized by three-thymine loops, similar to the substrate used for the crystallization of T7 endonuclease I (7). From our crystallization screens, we obtained crystals of protein alone and in complex with the DNA. The protein-alone structure was solved at 1.35 Å resolution using SAD methodology (Table 1). While this work was underway, an unliganded structure of Tt-RuvC was reported by Chen *et al.* (21). The orthorhombic crystal form obtained by these authors is the same as the one grown by us, and the structure reported herein is virtually identical to the published one. Tt-RuvC is also very similar to *E. coli* RuvC (20). When the structures of the two proteins are superimposed using the positions of 126 C-α atoms of one subunit of the dimer, the resulting root-mean-square deviation (rmsd) is only 1.2 Å. The core of the RuvC structure is formed by a five-stranded β-sheet made of three anti-parallel strands and two parallel strands—a characteristic feature of the RNase H fold (11). On one side of the β-sheet are two helices (A and B) that participate in dimer interface formation. On the other side are three helices: two short helices (C, D) and a longer helix (E) that runs across the face of the β-sheet.

**Figure 1.**
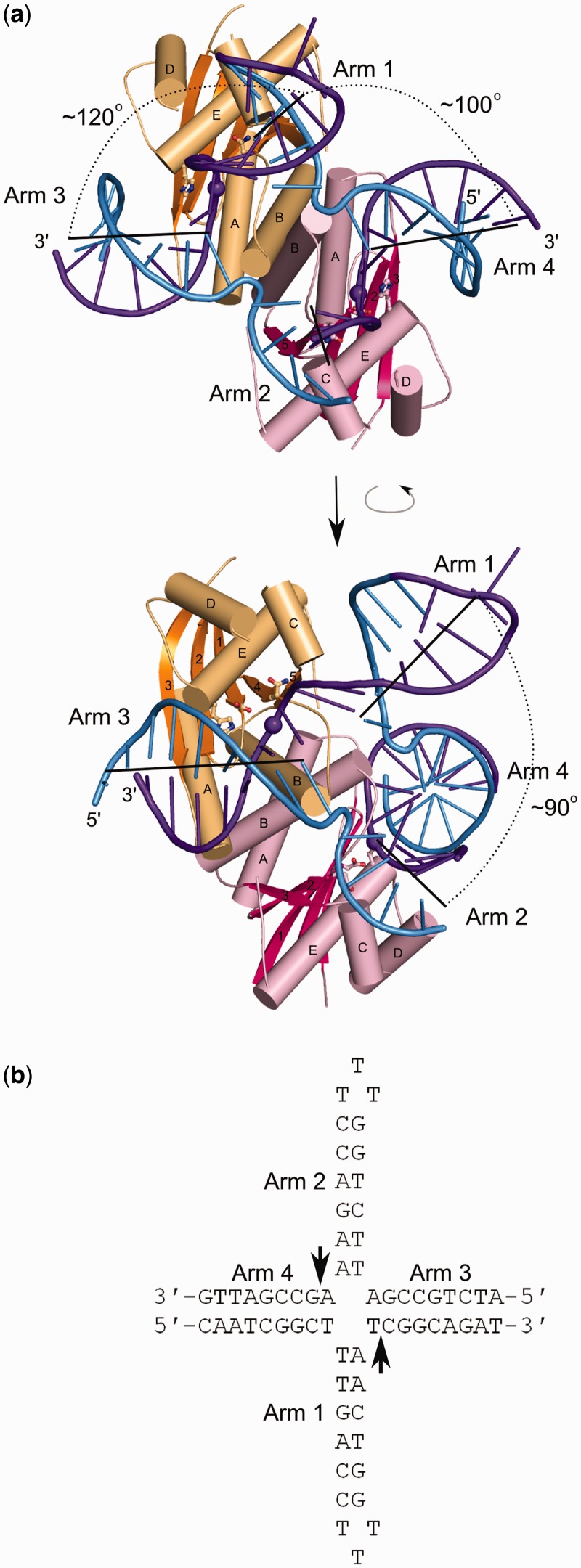
Structure of Tt-RuvC in complex with a synthetic HJ. (**a**) Two views of the structure. The two subunits of the protein dimer are shown in orange and pink, with the central β-sheet in a darker color. The secondary structure elements are labeled. The DNA is shown in ladder representation with cleaved strands in darker color and the scissile phosphates shown as large spheres. The active-site residues are shown in a ball-and-stick representation. (**b**) Sequence of the HJ present in the crystal structure. The phosphates located in the vicinity of the active sites are indicated with arrows.

One crystal form obtained in our crystallization screens contained both the protein and DNA. These crystals had a long-rod shape and were regular but diffracted X-rays very weakly. After testing >1500 crystals at three synchrotron beamlines, we collected four data sets, all at ∼3.8 Å resolution. All of our analyses are based on the best data set that extended to 3.75 Å (Table 1). The structure was solved using MR with the apo protein structure and short fragments of ideal B-form DNA as the search models. The asymmetric unit contains a complex made of the RuvC dimer and the HJ and MR Phaser software (23) readily found the solution for the protein dimer and two or three arms of the HJ, depending on the data set selected. The arms consistently found for all MR runs were the two cleaved arms three and four (see later in the text). Importantly, the positioning of three HJ arms in the complex was not imposed in any way but was independently determined by the MR method. The only arm that was not properly located was arm 2, which does not form any crystal contacts and is the least ordered and only partially built in the structure. The ends of the other three arms are stabilized by contacts with symmetry-related protein molecules (Supplementary Figure S2). The complete model of the complex was subsequently built manually and refined (Figure 1; for details, see Supplementary Materials and Methods). The 2Fo-Fc electron density maps after refinement are shown for the DNA model in Supplementary Figure S3a.

To further verify our model and minimize errors in the placement of complex components that result from model bias at low resolution, we calculated two types of omit maps for the DNA. The first type was a composite simulated annealing (SA-omit) map. For the second type, we prepared a bias-free model by superimposing the unliganded form of Tt-RuvC on the protein subunits in the final complex structure and fragments of ideal B-form DNA on the HJ arms with small rigid body adjustments of the positions of individual nucleotides. We then removed groups of three to four DNA nucleotides from the HJ model and refined the resulting structures in Phenix. Fo-Fc maps for the omitted nucleotides were combined, resulting in an omit map for the entire DNA. The two types of omit maps are shown in Supplementary Figure S3b and c, and they confirmed the correctness of the placement of the DNA model in the structure.

In the Tt-RuvC–HJ structure, the DNA is located in the vicinity of both active sites; therefore, the observed arrangement is consistent with a productive configuration. However, the RuvC preferred cognate sequences introduced into the oligonucleotides are located in the DNA strands that do not interact with the active sites. Such substrate arrangement is most likely determined by crystal packing. RuvC shows sequence specificity in cleavage but not in binding (27), rendering such positioning of the DNA possible. Moreover, it has been shown that RuvC is able to cut sequences other than the cognate one, albeit with lower efficiency (19). The minimal requirement is a thymine on the 3′ side of the scissile phosphate—a configuration at one of the active sites in our structure, implying that we observe a catalysis-competent binding mode. Based on the structure, we also produced crystals with analogous HJs but with full preferred cognate sequences relocated to the regions that interact with the active site. Unfortunately, these crystals did not diffract X-rays.

### Conformation and binding of the DNA

The junction in complex with RuvC has the same non-crystallographic 2-fold symmetry as the protein dimer. When the two halves of the HJ are superimposed using the positions of 22 pairs of phosphorus atoms, the rmsd is 2.0 Å. The structure of the DNA can be described as tetrahedral. The arms are unstacked, and the two opposite arms located in the vicinity of the active sites are pointing downward from the plane between the HJ exchange point and the protein. We refer to these arms as ‘cleaved arms’ (labeled 3 and 4 in Figure 1). The two other arms (‘non-cleaved arms’; labeled 1 and 2 in Figure 1) are pointing upward, resulting in ∼90° angles between arms 1 and 2 and 120° angles between arms 1 and 3.

**Figure 2.**
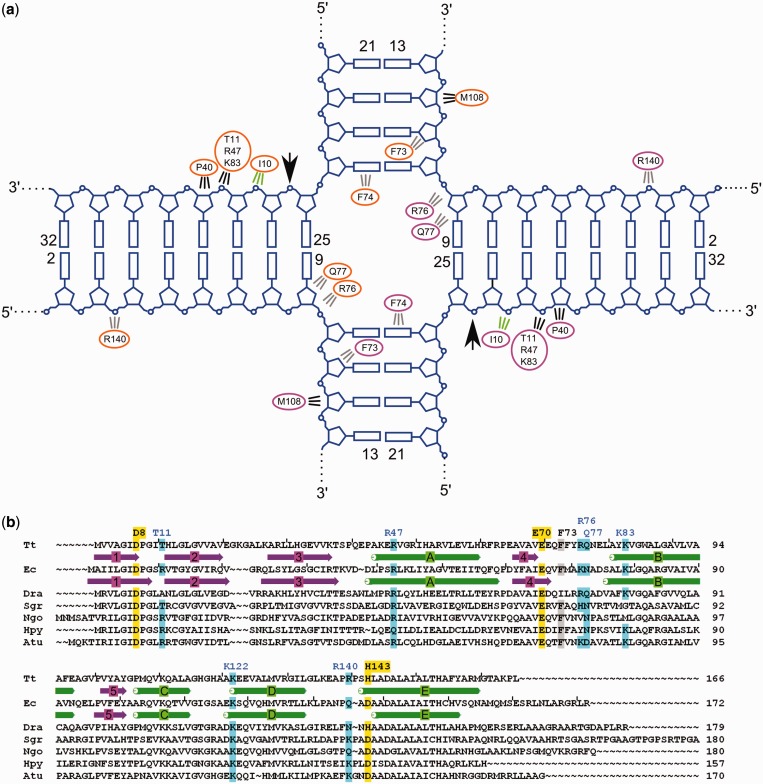
Substrate binding. (**a**) Schematic representation of substrate binding. Cleavage sites are shown with arrows, and the residues involved in binding are listed. Interactions shown in gray correspond to residues for which side-chain electron densities are not well defined, but based on the proximity of these residues to the DNA, they are predicted to form contacts with the substrate. Interactions in green are mediated by the backbone of the protein. (**b**) Multiple sequence alignment of RuvC proteins. The most divergent sequences were selected based on phylogenetic tree analysis and aligned. Tt, *T. thermophilus*; Ec, *E. coli*; Dra, *D.radiodurans*; Sgr, *Streptomyces griseus*; Ngo, *Neisseria gonorrhoeae*; Hpy, *Helicobacter pylori*; Atu, *Agrobacterium tumefaciens*. The residues that form the active site are highlighted in yellow. The residues that interact with the substrate (either observed or predicted) are highlighted in blue (charged interactions) and gray (van der Waals interactions). For sequences with known structures β-strands and α-helices are shown in purple arrows and green rectangles, respectively. Tick marks are placed every 10 residues in Tt-RuvC and Ec-RuvC sequences.

RuvC approaches the scissile phosphates from the minor groove side of the double helix. The protein covers 4 bp on arms 1 and 2 and 8 bp on arms 3 and 4 (Figures 1 and 2), which is consistent with footprinting data (26). We observed weak electron densities for the side chains of M108 and K83 involved in substrate binding; for other residues, we predicted the interaction based on their vicinity to the DNA substrate and using the high-resolution unliganded structure as a guide (Figure 2a). This is aided by the fact that RuvC does not undergo any major conformational changes on substrate binding with the exception of the loops stabilizing the exchange point. Seven DNA-binding residues have also been verified in cross-linking studies (see later in the text).

The exchange point of the junction is stabilized by the *N*-termini of helices B from each subunit, which also participate in dimer formation (Figures 1 and 2a). Loops preceding helices B are located in the HJ opening. In the absence of the substrate, these loops are highly mobile and can adopt different conformations in each subunit of the RuvC dimer (20,21). It has been proposed that this asymmetry allows only one cleavage of HJ to occur at a time (21). However, in our complex structure, the loops from both dimer subunits adopt a similar conformation, suggesting that a different mechanism is responsible for sequential cleavages by RuvC.

The loops can form several interactions with the DNA. For example, although the electron density for the side chain of R76 is not clearly defined, the location of this residue could allow its side chain to be inserted into the opening of the HJ. The importance of R76 is further demonstrated by the fact that the R76A variant of Tt-RuvC protein had reduced activity (Supplementary Figure S1). For *E. coli* RuvC, the effect of substituting the lysine equivalent of R76 was studied *in vivo.* By itself, this substitution did not produce ultraviolet-light sensitivity (28), but it did decrease ultraviolet-irradiation survival of bacteria when combined with a substitution of the equivalent of Tt-RuvC T11. The *E. coli* enzyme is a more robust enzyme than Tt-RuvC (Supplementary Figure S1), which is the likely reason why multiple substitutions are needed to affect its function. One difference that is apparent from the alignment of RuvC sequences is the Y75 residue in the loop before helix B in Tt-RuvC, which is replaced by an alanine in the *E. coli* enzyme. This substitution could explain the lower activity of the *T. thermophilus* enzyme. In both the unliganded structure and the HJ complex, we observed electron density for the side chain of Y75. The loop and Y75 undergo a large conformational change on substrate binding, and the presence of the bulky tyrosine side chain may hinder these changes, lowering protein’s activity. Indeed, when Y75A substitution was introduced to Tt-RuvC, the activity of the enzyme was markedly increased (Supplementary Figure S1)—an effect also observed by Chen *et al.* (21).

Another substrate interface residue is the conserved F73 located close to the cross-over point. Even though we do not observe the electron density for its side chain, it could form a flat hydrophobic interface for binding the non-cleaved strand (Figure 2). The F-to-A substitution in the *E. coli* enzyme affected substrate binding (29) and for both *E. coli* and *T. thermophilus* RuvC reduced the activity (21,29). Additional residue positioned to potentially interact with the DNA is K122, which could bind the exchange-point phosphate in the cleaved strand. The amine group of nearby K111 is further away from the exchange point phosphate but could form a water-mediated interaction with this group. Both K111 and K122 are strongly conserved evolutionarily (Figure 2b), and substitution of the corresponding residues in the *E. coli* enzyme led to a reduction of substrate cleavage (28). An important protein–DNA interaction occurs between the phosphate of the cleaved strand 2 nt from the active site toward the 3′-end. This phosphate is positioned to interact with T11, R47 and K83. Residues that correspond to R47 and K83 have been substituted in *E. coli* RuvC, which led to defects in junction binding and cleavage (30). In our structure, the non-cleaved strand is bound by R140 (Figure 2a).

The first two active site residues, aspartate and glutamate, are strictly conserved in RuvC proteins from various species (Figure 2b), and the third residue is an aspartate in most species. In *Deinococcus-Thermus* phylum, which comprises *T. thermophilus* and *Deinococcus radiodurans*, the third residue is a histidine. When we replaced the histidine with aspartate in Tt-RuvC (H143D variant), the activity of the enzyme was increased and a double substitution Y75A/H143D was even more active, nearly matching the *E. coli* enzyme (Supplementary Figure S1). Therefore, *Deinococcus-Thermus* phylum RuvC proteins may have evolved to be less active by the introduction of a bulky residue in the loop stabilizing the exchange point (tyrosine in *Thermus* and arginine in *Deinococcus*) and changing the last aspartate of the active site to a histidine. Perhaps this is due to the fact that these bacteria are polyploid (31), and to survive in harsh conditions, they rely heavily on homologous recombination. The lower activity of RuvC protein may facilitate the regulation of this process.

### Chemical cross-linking

To further verify the protein-substrate contacts and positioning of the HJ observed in our structure, we performed chemical cross-linking studies using a method developed by Verdine and co-workers (32,33). In this method, a thiol group on a two-carbon tether is specifically introduced to the nucleic acid, and a cysteine residue is introduced to the protein via site-directed mutagenesis. A disulfide bond is formed between them only when the thiol groups from the protein and the substrate are positioned in close proximity on complex formation, resulting in a covalent adduct.

**Figure 3.**
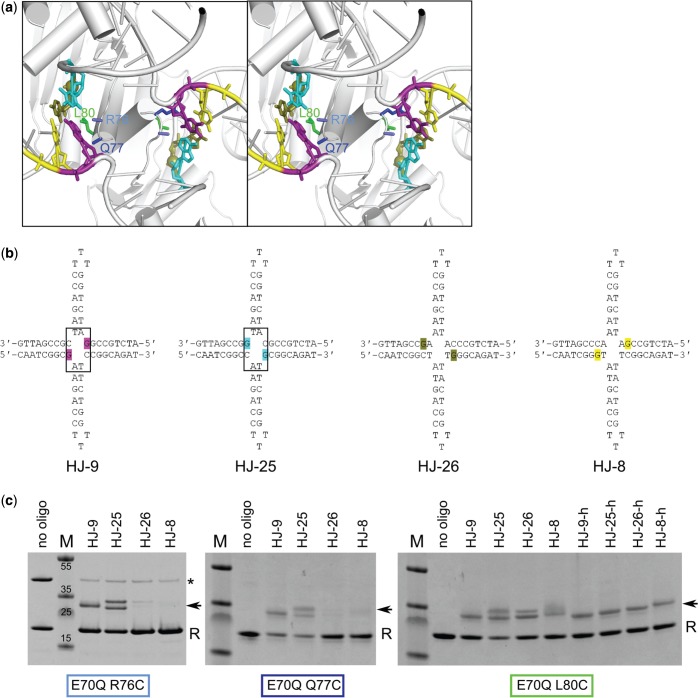
Chemical cross-linking with base-modified HJs. (**a**) Stereoview of a close-up of the HJ exchange point. The residues that are substituted with cysteines and the nucleotides with modified bases are shown as colored sticks and labeled light blue for R76, dark blue for Q77 and green for L80. Nucleotides with modified bases are shown in purple, cyan, yellow and olive. (**b**) Sequence and structure of modified HJs used in cross-linking experiments. The site of modification is highlighted in the same colors as in panel (a). (**c**) Cross-linking reactions. The cysteine variants indicated at the bottom of each gel were mixed with modified HJs given on top of each lane, and the reaction products were visualized in Coomassie-stained denaturing PAGE. For the L80C variant, HJs with only one modification were tested and are labeled from HJ-8-h to HJ-26-h. The RuvC band is marked with ‘R’. The covalent RuvC dimer is indicated with an asterisk, and cross-linking products are indicated with arrows. The molecular weight markers are in lanes denoted with ‘M’ and labeled.

We first used oligonucleotides in which a thiol group was attached to the amine group in position 2 of the aromatic ring of guanine, which is located in the minor groove in the double helix. In our structure, the minor groove edges of the bases of nucleotides 8, 9, 26 and 25 are in the vicinity of the *N*-terminal regions of helices B and the loops that precede them. Three protein residues from this region are located close to the DNA: R76, Q77 and L80 (Figure 3a). We prepared HJs with the same structure and arm lengths as the ones used for crystallization but with thiol groups symmetrically attached to the bases of both copies of nucleotides 8, 9, 26 and 25 (HJ-8, HJ-9, HJ-25 and HJ-26, respectively; Figure 3b). The DNA sequence was changed to incorporate guanines in these four positions. Oligonucleotides HJ-9 and HJ-25 were designed to contain a 2 bp homology core, allowing the modified base to be located 1–2 nt on either side of the exchange point (Figure 3b). We reasoned that such mobility could facilitate the proper positioning of these bases for cross-linking. To further verify the specificity of the reaction, we designed oligonucleotides HJ-8 and HJ-26, which did not have the homology core, leading to immobile modified nucleotides located 2 nt from the exchange point. We then prepared Tt-RuvC variants with inactivating active site substitution E70Q and cysteine substitutions of R76, Q77 or L80. The wild-type sequence does not contain any cysteines; therefore, the introduced ones were the only ones present. Cysteine variants of Tt-RuvC were mixed with modified oligonucleotides, and the resulting cross-linking products were analyzed on sodium dodecyl sulfate-polyacrylamide gel electrophoresis (SDS–PAGE) gels. As predicted from the structure, Tt-RuvC R76C and Q77C formed cross-linked complexes with high efficiency with HJ-9 and HJ-25. Very little product was formed when R76C and Q77C were mixed with HJ modified in nucleotides 8 and 26 (Figure 3c). This agrees with the crystal structure because bases of nucleotides 8 and 26 are too far from R76 to form a cross-link. The observed weak reactivity of R76C toward HJ-26 and HJ-8 can be explained by a migration of the exchange point, which would result in a less stable HJ with mismatches in HJ-26 and HJ-8. L80 is equidistant to all four bases modified in our experiments. As expected, all of the tested oligonucleotides efficiently reacted with the L80C variant (Figure 3c). As a negative control, Tt-RuvC R76C and L80C did not react with the HJ with thiol modification of the phosphate backbone described later in the text (Supplementary Figure S4a).

In reactions with HJ-25 and HJ-26, the cross-linked species migrated as two bands. We reasoned that the lower band can result from the attachment of a single RuvC molecule to the DNA and the upper band from the attachment of two protein molecules. This notion was corroborated in experiments in which cross-linking of the L80C variant was tested with HJs with only a single modified base (HJ-8-h, HJ-9-h, HJ-25-h and HJ-26-h). Only the lower band was observed in these experiments, confirming the attachment of a single protein molecule (Figure 3c). HJ-25 and HJ-26 contained the modification in the strand that interacts with the active site, which is stably bound. This is the likely reason why cross-linking with both subunits of the RuvC dimer occurs. In the experiments with HJ-8 and HJ-9, a single protein adduct was formed, possibly because the cross-linking with the first protein residue resulted in small asymmetry in the HJ that prevented cross-linking with the second protein subunit. We noted that the cross-linked species migrate faster than would be expected based on their molecular weight, but we assume that their mobility is affected by the high negative charge of the HJ and the fact that its structure remains intact in SDS–PAGE (see later in the text).

**Figure 4.**
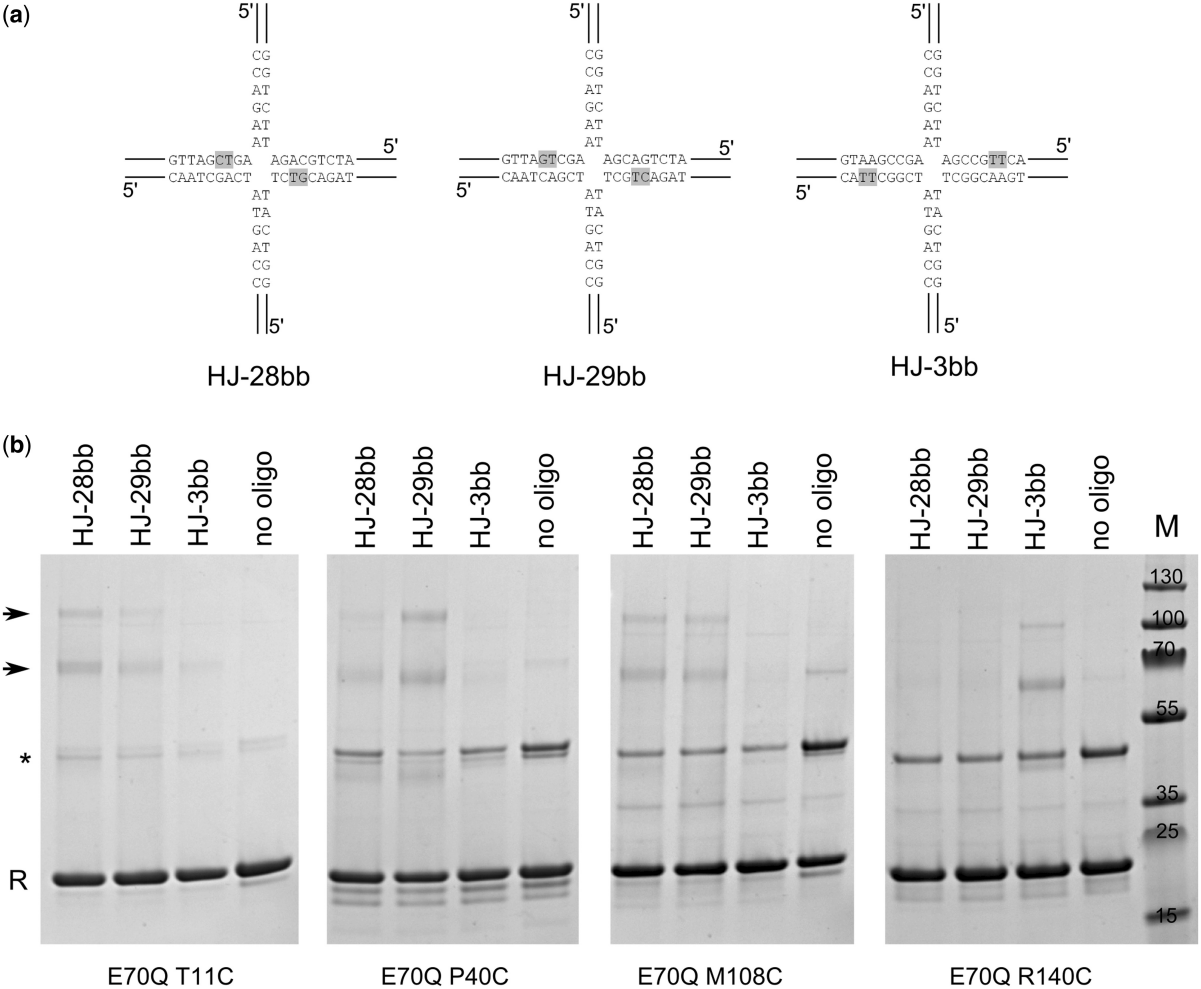
Chemical cross-linking with backbone-modified HJs. (**a**) Sequence and structure of backbone-modified HJ used in cross-linking experiments. Modified phosphate groups were located between two nucleotides highlighted in gray. (**b**) Cross-linking reactions. The cysteine variants indicated at the bottom of each gel were mixed with modified HJs given on top of each lane, and the reaction products were visualized in Coomassie-stained denaturing PAGE. The RuvC band is marked with ‘R’. The covalent RuvC dimer is indicated with an asterisk, and cross-linking products are indicated with arrows.

To probe the interactions between the protein and backbone of the substrate, we prepared HJ substrates in which one of the non-bridging oxygens of the phosphate group of the DNA was replaced by an amine group with a two-carbon tether and thiol group (32). These HJs had an immobile exchange point and longer 40 bp arms. We increased their length to move the modification sites further from the arms’ ends. We attempted to prevent the cross-linking of non-productive complexes that could form if the ends of the arms that contain the modification bound to the interface for cleaved arms 3 and 4, positioning the exchange point outside the protein dimer. Thiol groups were symmetrically introduced to two copies of the phosphate groups in positions 3, 28 and 29, resulting in oligos HJ-3bb, HJ-28bb and HJ-29bb, respectively (Figure 4a; although these HJs were longer, we used the same numbering as in Figures 2 and 3 for consistency). In the Tt-RuvC-HJ structure, the phosphate of nucleotide three is in the vicinity of R140, and the phosphates of nucleotides 28 and 29 are located close to T11 and P40, respectively (Figure 2a). After HJ, arms corresponding to 3 and 4 in the complex structure are exchanged with arms corresponding to 1 and 2 (Figure 1)—an equally probable binding mode, the phosphate group of residue 28 is in the vicinity of M108. We tested the cross-linking between the backbone-modified DNAs and Tt-RuvC E70Q variants with T11C, P40C, M108C or R140C substitution (Figure 4). The efficiency of these cross-linking reactions was lower compared with the base-modified DNAs, which is at least partially attributable to the fact that the attachment of the thiol to the phosphate group results in two stereoisomers, only one of which is able to react with the protein. As expected, the T11C variant reacted most efficiently with HJ-28bb, but some product was also observed for HJ-29bb, possibly resulting from the migration of the exchange point which would lead to mismatches. Similarly, HJ-29bb reacted most efficiently with P40C and to a lesser extent with T11C (Figure 4b). Tt-RuvC M108C reacted with both HJ-28bb and HJ-29bb. We expected more efficient adduct formation with HJ-28bb, and the cross-linking with both HJ-28bb and HJ-29bb can result from either the exchange point migration or flexibility of this part of the interface. Indeed, the non-cleaved arms form very few contacts with the protein (Figure 2a). As predicted based on the Tt-RuvC-HJ structure, the R140C variant reacted only with HJ-3bb, and no product was observed for HJ-28bb or HJ-29bb (Figure 4b).

Similar to the reactions with base-modified DNAs, the cross-linking with backbone-modified oligonucleotides also resulted in two products with higher and lower mobility. Therefore, we again used oligonucleotides with single modifications to establish the identity of these bands. We first verified whether HJ themselves were migrating as multiple bands on SDS–PAGE (Supplementary Figure S4b). For symmetrically (doubly) modified HJs, the gradual addition of the four strands resulted in single bands with stepwise-reduced mobility, confirming that the four-way structure was stable and migrated as a single band. However, for DNAs with single modification, the DNA migrated as two bands. On the addition of the Tt-RuvC T11C variant to asymmetrically modified HJ-29bb-h or the P40C variant to HJ-28bb-h, the pattern of cross-linked products corresponded to the DNA alone but with lower mobility that resulted from attachment of the protein. For symmetrically modified DNA, two products were observed, although the DNA alone migrated as a single band on SDS–PAGE, most likely resulting from the attachment of two protein molecules. The formation of the cross-linked products with two RuvC molecules that were attached for both base- and backbone-modified DNAs confirmed the symmetrical interaction of the RuvC dimer with the substrate observed in our structure. In summary, the results of the cross-linking experiments correspond very well with our structure and confirm that the protein–DNA contacts we observe occur also in solution.

### Comparison with other resolvases

**Figure 5.**
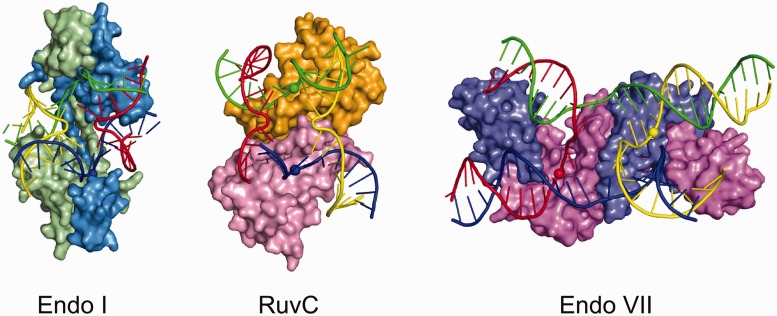
Comparison of known DNA complex structures of HJ resolvases: endonuclease I (PDB: 2PFJ), RuvC and endonuclease VII (PDB: 2QNC). Proteins are shown in surface representation with two different colors for each dimer subunit. The substrate is shown in a cartoon representation and colored as in (34). Scissile phosphates are shown as spheres. Notice that the endonuclease I structure interacts with the opposite side of the junction, and the structure is shown rotated 180° around a vertical axis.

The other available structures of resolvase-substrate complexes are those of two phage enzymes: endonuclease I from bacteriophage T7 and endonuclease VII from bacteriophage T4 (7,34) (Figure 5). The two proteins and RuvC belong to different classes of nucleases and have different folds. Endonuclease VII adopts a β-β-α-Me fold, and endonuclease I belongs to the PD-(D/E)XK nuclease family. Both enzymes form dimers. In contrast to RuvC, however, they adopt highly intertwined dimeric structures. The three enzymes exhibit striking differences in substrate binding and cleavage (Figure 5). In the endonuclease VII complex structure, the three arms of the HJ are almost in one plane, and one is pushed up from the plane by ∼25°. The situation is different in RuvC and endonuclease I, in which the two halves of the junction are at almost a 90° angle. In endonuclease I, two arms in each half of the junction are essentially co-axial, whereas both halves are bent at the exchange point in RuvC. RuvC and endonuclease VII approach the scissile phosphate from the minor groove side and endonuclease I from the major grove side. Both endonuclease I and RuvC cleave 1 nt away from the exchange point, but the site of cleavage is different. For RuvC, the scissile phosphate is located toward the 3′-end of the cleaved strand. For endonuclease I, the scissile phosphate is located toward the 5′-end. For endonuclease VII, the cleavage site is located 2 nt from the exchange point toward the 3′-end of the cleaved strand.

RusA is another bacterial resolvase of phage origin. Like RuvC, it has been shown to bind the HJ in a tetrahedral conformation (35,36). However, based on the RusA structure in complex with two dsDNA fragments, a model was proposed that suggested that RusA exhibits yet another HJ binding mode in which the center of the HJ is open with unpairing of the bases to accommodate loop regions of the protein (37). Altogether, comparisons with other enzymes show that RuvC exhibits a new type of HJ recognition.

RuvC is more specific than phage resolvases—it cleaves only HJ substrates. The protein–DNA interactions appear to be much more extensive in phage enzymes, in particular in endonuclease VII. Therefore, the presence of fewer DNA arms, for example in the three-way junctions, would not dramatically reduce the affinity for the substrate. With fewer contacts in RuvC, all the interactions may be important, leading to binding and cleavage of only four-way junctions.

## DISCUSSION

In this work, we present the first crystal structure of a substrate complex of RuvC resolvase, which was solved at 3.75 Å resolution. This is moderate resolution for crystal structures. Although a high-resolution structure of the protein alone was used to solve it, fine details of our structural model have to be treated with caution. This applies in particular to the loops that stabilize the exchange point of the HJ, which change their conformation on substrate binding. To further corroborate the DNA arrangement and protein–DNA contacts, we performed cross-linking experiments using site-specific thiol-based methods. The results of these experiments are consistent with the crystal structure. Moreover, the structure is in accordance with mutational data available in the literature.

Our structure visualizes the global arrangement of HJ DNA in complex with a bacterial resolvase. This configuration is strikingly different from the phage enzymes studied to date, demonstrating that various modes of HJ recognition evolved. Our structural model also provides information about the cleavage site relative to the exchange point. Additionally, we show that the exchange point of the HJ is stabilized by the *N*-termini of helices B, which also participate in protein dimer formation.

Among the most important insights from our structure is the determination of the conformation of the HJ in complex with RuvC. Electrophoretic mobility and Förster resonance energy transfer experiments showed that the junction becomes 2-fold symmetrical on binding by RuvC but with unstacked arms (26,38). This is consistent with the tetrahedral conformation of the HJ observed in our structure. This conformation also explains the results from and Förster resonance energy transfer studies, which showed a large separation of the ends of the four arms of the junction upon RuvC binding (38). The conformation of the DNA observed in our RuvC complex structure is different from the square planar conformation adopted by the HJ in complex with RuvA. The two proteins clearly cannot interact effectively with the DNA at the same time, and a conformational change of the substrate is needed when it is handed from RuvA to RuvC.

The location of the scissile phosphate in the RuvC-DNA complex was unclear from previous studies (19). In a study by Fogg *et al.* (27), the most efficient cleavage of HJs with immobile crossover points was reported to occur exactly at the exchange point. Experiments performed with substrates that contained two crossover points, one of which was immobile, showed that the cleavage occurred 1 nt from the exchange point toward the 3′-end of the cleaved strand (39). The location of the scissile phosphate in our structure (Figures 1 and 2) is consistent with the cleavage data reported by Sha *et al.* (39). The more efficient cleavage at the exchange point reported by Fogg *et al.* (27) can be explained by the migration of the branch point by 1 bp to position the cognate sequence at the active site, resulting in the formation of mismatches in the cleaved arms. One important question that remains unanswered is the mechanism that underlies the sequence preference of RuvC, which likely relies on subtle differences in the interactions between particular bases and the protein.

In conclusion, our structure provides the first structural information about the mechanism of HJ recognition by cellular resolvases. It also broadens our understanding of the possible modes of HJ binding by resolvases and provides important information about the overall conformation of DNA.

## ACCESSION NUMBERS

The atomic coordinates of the Tt-RuvC-HJ complex were deposited in the Protein Data Bank (accession code 4LD0).

## SUPPLEMENTARY DATA

Supplementary Data are available at NAR Online, including [40–45].

## FUNDING

Wellcome Trust International Senior Research Fellowship [081760 to M.N.]; Recipient of Foundation for Polish Science ‘Ideas for Poland’ award (to M.N.); The access to ESRF was financed by the Polish Ministry of Science and Higher Education [project no. ESRF/73/2006]; The research leading to these results has also received funding from the European Community’s Seventh Framework Program under grant [agreement no. 226716]. Funding for open access charge: Wellcome Trust Senior Research Fellowship.

*Conflict of interest statement*. None declared.

## Supplementary Material

Supplementary Data
