# Supplementary material for: Crystal structure of RuvC resolvase in complex with Holliday junction substrate
Source: Nucleic Acids Res. 2013 Aug 24;41(21):9945–55. doi: 10.1093/nar/gkt769 (PMC3834835; doi:10.1093/nar/gkt769)
Supplement: Supplementary Data [file supp_41_21_9945__index.html]

Crystal structure of RuvC resolvase in complex with Holliday junction substrate — Crystal structure of RuvC resolvase in complex with Holliday junction substrate — Supplementary Data 

# Crystal structure of RuvC resolvase in complex with Holliday junction substrate

## Supplementary Data

files

**Files in this Data Supplement:**

- Supplementary Data - pdf file
