# Supplementary material for: Crystal structure of RuvC resolvase in complex with Holliday junction substrate
Source: Nucleic Acids Res. 2013 Aug 24;41(21):9945–55. doi: 10.1093/nar/gkt769 (PMC3834835; doi:10.1093/nar/gkt769)
Supplement: Supplementary Data [file supp_gkt769_nar-01770-h-2013-File007.pdf]

Crystal structure of RuvC resolvase in complex with Holliday junction substrate

Karolina M. Górecka, Weronika Komorowska, and Marcin Nowotny

SUPPLEMENTARY INFORMATION

MATERIALS AND METHODS

Protein preparation

T. thermophilus RuvC (Tt-RuvC) expression plasmids were prepared based on the pET28 expression vector (Merck KGaA, Darmstadt, Germany). The sequence coding for the protein was inserted into NdeI and EcoRI restriction sites, and expressed protein contained an *N*-terminal His-tag. Mutagenesis of the construct was performed using the QuikChange kit (Agilent Technologies, Santa Clara, CA, USA).

Wildtype and substitution variants of Tt-RuvC proteins were expressed in the *E. coli* BL21 strain using induction with 0.4 mM isopropyl β-D-1-thiogalactopyranoside. Bacterial cells were resuspended in 40 mM NaH_2PO_4 (pH 7.0), 75 mM NaCl, 5% glycerol, and 1.4 mM β-mercaptoethanol, with the addition of a mix of protease inhibitors. Lysozyme was then added to a final concentration of 1 μg/ml. After incubation on ice for 30 min and sonication, imidazole was added to the supernatant to a final concentration of 10 mM, which was applied to a Nickel column (GE Healthcare) equilibrated with 10 mM imidazole, 40 mM NaH_2PO_4, 500 mM NaCl, and 5% glycerol. The protein was eluted with a gradient of imidazole from 10 to 300 mM, and the fractions that contained the protein were dialyzed overnight in a buffer that contained 40 mM NaH_2PO_4, 75 mM NaCl, 5% glycerol, 0.1 mM dithiothreitol (DTT), and 0.5 mM ethylenediaminetetraacetic acid (EDTA). His-tagged protein was used for the

biochemical assays. For crystallization, the tag was removed with PreScission protease, and protein was further purified on a Mono-S column (GE Healthcare). It was eluted with a linear gradient of NaCl from 75 to 500 mM and concentrated to 16-26 mg/ml. Tt-RuvC was stored in 20 mM HEPES (pH 7.0), 100 mM NaCl, 5% glycerol, 0.1 mM DTT, and 0.5 mM EDTA. Selenomethionine-labeled (SeMet) Tt-RuvC (E70Q) was expressed using methionine auxotrophic *E. coli* strain B834 (DE3) pLysS in a defined medium (40) and purified using the same protocol.

Crystallization and structure solution

Unmodified high-performance liquid chromatography-purified oligonucleotides were purchased from Metabion International AG (Martinsried, Germany). The sequences are given in Supplementary Table 1. For the crystallization experiments, Tt-RuvC E70Q (7 mg/ml final concentration) was mixed with the DNA at a 1.8:1 molar ratio, and $MgCl_2$ was added to a final concentration of 5 mM. The complexes were mixed with the reservoir solution at equal volume and crystallized by the sitting drop vapor diffusion method at 18°C. The content of the crystals was analyzed on polyacrylamide gels (sodium dodecyl sulfate-polyacrylamide gel electrophoresis [SDS-PAGE] for protein and Tris/Borate/EDTA-urea PAGE for nucleic acid).

Crystals of the apo protein were obtained with 0.1 M citric acid, pH 3.5, and 1.5 M NaCl. Interestingly, we did not observe the growth of apo protein crystals if DNA was absent in the mixture, indicating that the DNA facilitated crystallization. For data collection, the crystals were transferred in two steps to a cryoprotecting solution with a 20% final glycerol concentration. All of the crystals were flash-frozen in liquid nitrogen. The diffraction data were collected with the Berliner Elektronenspeicherring-Gesellschaft für Synchrotronstrahlung synchrotron at beamline MX-14.2 on a Mar225 CCD detector at 100 K (41). The datasets were processed and scaled using XDS (42) (Table 1). The apo structure was

first solved using selenomethionine single-wavelength anomalous diffraction and SOLVE/RESOLVE (43). The structure was refined to 1.35 Å resolution (Table 1) using phenix.refine (24) interspersed with manual building in Coot (44). One protein dimer is present per asymmetric unit, and the two subunits are very similar, differing only in the conformation of a loop located before helix B, indicating the mobility of this region in the absence of the DNA. This structure was used as a search model to solve the complex structure and as a reference model during its refinement. It was not deposited in the PDB, since the same structure at slightly higher resolution became available when this work was underway (PDB ID: 4EP4) (21).

The crystals of the productive Tt-RuvC-DNA complex were obtained with oligos J221 and J222 and J221m/J222m, which mimic the HJ that contains a 2 bp homology region in the center. This allows the crossover point to migrate by 1 bp and form a HJ with 10 and 5 bp arms or 9 and 6 bp arms. The arm lengths were chosen based on published footprinting studies (26). The shorter arms contained three thymine loops at the ends to stabilize them, similar to the substrate used to solve the endonuclease I complex structure (7). The crystals were grown in 0.3-0.5 M ammonium phosphate. They were large and regular but diffracted X-rays to only ~7 Å resolution. To improve X-ray diffraction, numerous modified synthetic HJs were used for crystallization (overall, 46 HJ variants were used during crystal optimization), but these efforts were unsuccessful. We next conditioned the original crystals by step-wise exchange of the well and drop solution with buffers with increasing ammonium phosphate concentrations. The final solution contained 0.7 M ammonium phosphate and 30% glycerol for cryoprotection. After such treatment and testing a large number of crystals at different beamlines, we were able to collect 3.75 Å resolution datasets at microfocus beamline 23-2 of the European Synchrotron Radiation Facility. The data were collected at 100 K on a Mar225 CCD detector. The collection of these data required the use of a microfocus beam and

a helical data collection strategy with translation along the crystal that was simultaneous with its rotation.

The structure was solved using molecular replacement with Phaser (23) and using the 1.35 Å apo protein structure and ideal B-form DNA as search models. Two or three DNA fragments that corresponded to HJ arms were found by MR software. Therefore, their position was not imposed in any way during initial model building. The use of the apo protein structure was also very effective because RuvC undergoes little conformational change upon substrate binding. The complete model of the complex was built in Coot and refined in phenix.refine (version 1.8.2-1309) (24) with rounds of manual building (Table 1). Base-pairing restraints were imposed during refinement, and the portion of the high-resolution unliganded protein structure that does not undergo conformational changes upon DNA binding was used as a reference model. Non-crystallographic symmetry (NCS) restraints between subunits of the protein dimer were imposed. We did not impose NCS for the DNA because the nucleotide sequences of the symmetry-related arms were different. In addition, although the two halves of the HJ have very similar structure they do not superimpose perfectly (rmsd of the positions of 22 pairs of phosphorus atoms is 2.0 Å). The entire dataset was merohedrally twinned with twin law –h, -k, l and ~15% twin fraction. However, twinning was not included in the refinement protocol because it resulted in much higher R factors. One B-factor per residue was refined. For the two oligos that formed the HJ, 31 and 22 nucleotides of 33 were observed in the electron density maps. Only one T_3 loop was traced, which is sandwiched between two symmetry-related protein molecules that form an important crystal contact (Supplementary Fig. 2). The other T_3 loop located in arm 2 had very poorly defined electron density maps and could not be traced.

The substrate used for crystallization is two-fold symmetric in its structure but not in the sequence. Therefore, two orientations that are not equivalent in sequence are possible but

cannot be distinguished because of the limited resolution of the structure. A combination of the two orientations may also be present in the crystal. Only one arbitrarily chosen orientation was built in the current model. Structural analyses, including superimpositions, were calculated in Pymol (http://www.pymol.org; accessed June 6, 2013). Figures were prepared using Pymol. Composite simulated annealing omit maps were calculated using CNS (Crystallography and NMR System) (45). One percent of the model was omitted at each step, and torsion angle molecular dynamics were performed at 1500 K.

RuvC cleavage assay

The cleavage assays were performed essentially as previously described (25), and the initial experiments were performed using the same oligonucleotides. They form synthetic junctions with 25 bp arms and contain two cognate sequences: TTTG in the middle of one cleaved strand and 5′-ATTC in the other. We sought to verify whether Tt-RuvC had the same sequence specificity as the *E. coli* enzyme, and we tested the activity of *T. thermophilus* protein with four different substrates, in which both cognate sequences were the same (ATTC, TTTC, ATTG, and TTTG). All of the sequences were cleaved with the same efficiency, so the results for the substrate with the TTTC cognate sequence are presented as representative (see Supplementary Table 1 for sequences, HJ4 5′-labeled with HEX dye). The standard cleavage reaction mixture (10 μl) contained RuvC and the substrate in molar ratios of 20:1, 10:1, 5:1, and 1:1 (4 pM of substrate in each sample and increasing concentration of enzyme). The reaction buffer contained 20 mM bicine (pH 9.0), 75 mM NaCl, 1 mM DTT, 100 μg/ml bovine serum albumin, 5% glycerol, and 5 mM Mg acetate. The samples were incubated for 1 h at 37°C for *E. coli* RuvC and 2 h at 65°C for *T. thermophilus* enzyme. The reaction was stopped by the addition of sample buffer that contained 20% glycerol, 1% SDS, 0.1 M EDTA (pH 8.0), and 0.1% bromophenol blue. The hydrolysis products were analyzed by 10% or

12% PAGE in Tris-acetate-EDTA buffer. 50-mer dsDNA that was 5'-labeled with HEX (oligonucleotides D5 and HJ4cs; Supplementary Table 1) was used as the marker of the product. The reaction products were visualized with a FLA-7000 scanner (Fuji).

Crosslinking experiments

Two cross-linking methods were used: through DNA bases and through DNA backbone. The modified oligonucleotides used in these experiments are shown in Fig. 3b and 4a, and their sequences are given in Supplementary Table 1. For cross-linking through DNA bases, each modified HJ was composed of two annealed oligonucleotides. As a control, a set of four HJs that that contained a single modification was used. Oligonucleotides with 2-F-dI in the specific positions to be modified were obtained from Metabion International AG (Martinsried, Germany) and delivered on the synthesis columns, with all protective groups attached. The columns were washed with 3% dichloroacetic acid in 1 ml CH_2Cl_2 for deprotection and then washed three times with 1 ml CH_2Cl_2. After the washing step, 0.3 ml of X-linker solution that contained a 90-fold molar excess of cysteamine ($NH_2CH_2CH_2SH$) over DNA in 37.5% Et_3N was applied to each column and incubated for 18 h with occasional agitation. The filtrate was collected and dried on a speedvac, and 1 mL AMA (ammonia and 40% methylamine; 1:1) was added. The remaining oligonucleotide was washed from the column with 1 ml of AMA buffer and added to the sample. The samples were incubated for 20 h at 60ºC. Oligonucleotides were then purified on a DNAPac100 column (Thermo Scientific) at 65ºC with buffer that contained 4 M urea, 20 mM MES pH 6.5), and 0.2% acetonitrile with a linear gradient of $NaClO_4$ from 1 to 400 mM. Purified oligonucleotides were precipitated with ethanol and suspended in water. The backbone-substituted oligonucleotides were synthesized by Future Synthesis (Poznan, Poland). Synthesis of these

modified oligonucleodies was based on the H-phosphonate chemistry. Cysteine was condensated with diester H-phosphonate with subsequent oxidation in presence of CCl_4.

A standard cross-linking reaction mixture (15 µl) contained cysteine-substituted RuvC and the modified substrate in a 1:1 molar ratio. The reaction buffer contained 100 mM NaCl, 10 mM HEPES (pH 7.0), 0.5 mM DTT, 15% glycerol, and 5 mM $MgCl_2$ for base modifications or the same buffer with the addition of 100 mM KCl for backbone modifications. The samples were incubated for 2.5 h at 37°C, followed by overnight incubation at 25°C. The reaction was stopped by the addition of sample buffer (NuPAGE Sample Buffer, Invitrogen). The products of the crosslinking reaction were analyzed on precast gels (NuPAGE Bis-Tris Mini Gels 4-12%, Invitrogen) stained with Bio-Safe Coomassie Stain (Bio-Rad). The reaction products were visualized with an Image Quant LAS400 scanner (GE Healthcare).

Supplementary Table 1. Sequence of oligonucleotides used for biochemical experiments and crystallization

HJ-28-bb A	GACGGCCTCGCAATCGACTATGACCGAGCACGCGAGATGT
HJ-28-bb. B	ACATCTCGCGTGCTCGGTCATTCT=GCAGATGCGGAGTGAA
HJ-28-bb. C	TTCACTCCGCATCTGCAGATTCTGGCTGTGGCGTGTTTCT
HJ-28-bb. D	AGAAACACGCCACAGCCAGAAAGT=CGATTGCGAGGCCGTC
HJ-29-bb. A	GACGGCCTCGCAATCAGCTATGACCGAGCACGCGAGATGT
HJ-29-bb. B	ACATCTCGCGTGCTCGGTCATTCGT=CAGATGCGGAGTGAA
HJ-29-bb. C	TTCACTCCGCATCTGACGATTCTGGCTGTGGCGTGTTTCT
HJ-29-bb. D	AGAAACACGCCACAGCCAGAAAGCT=GATTGCGAGGCCGTC
HJ-3-bb. A	GACGGCCTCGCAT=TCGGCTATGACCGAGCACGCGAGATGT
HJ-3-bb. B	ACATCTCGCGTGCTCGGTCATTCGGCAAGTGCGGAGTGAA
HJ-3-bb. C	TTCACTCCGCACT=TGCCGATTCTGGCTGTGGCGTGTTTCT
HJ-3-bb. D	AGAAACACGCCACAGCCAGAAAGCCGAATGCGAGGCCGTC
HJ-28-bb/<br>HJ-29-bb B single	ACATCTCGCGTGCTCGGTCATTCTGCAGATGCGGAGTGAA
HJ-3-bb. A single	GACGGCCTCGCATTCGGCTATGACCGAGCACGCGAGATGT
HJ-9 1	CAATCGGCXAAGACCTTTGGTCTTCCGGCAGAT
HJ-9 2	ATCTGCCGXATCTGGTTTCCAGATCGCCGATTG
HJ-25 1	CAATCGGCCAAGACCTTTGGTCTTXCGGCAGAT
HJ-25 2	ATCTGCCGCATATGGTTTCCAGATXGCCGATTG
HJ-26 1	CAATCGGCTATGACCTTTGGTCATTXGGCAGAT
HJ-26 2	ATCTGCCCATTCTGGTTTCCACAAAXCCGATTG
HJ-8 1	CAATCGGXTATGACCTTTGGTCATTCGGCAGAT
HJ-8 2	ATCTGCCXATTCTGGTTTCTAGAAACCCGATTG
HJ-9 2 single	ATCTGCCGGATCTGGTTTCCAGATCGCCGATTG
HJ-25 2 single	ATCTGCCGCATATGGTTTCCAGATGGCCGATTG
HJ-26 2 single	ATCTGCCCATTCTGGTTTCCACAAAGCCGATTG
HJ-8 2 single	ATCTGCCGATTCTGGTTTCTAGAAACCCGATTG
HJ-1-tttc	GGTAGGACGGCCTCGCAATCGGC**TTTC**ACCGAGCACGCGAGATGTCAACG
HJ-2-tttc	CGTTGACATCTCGCGTGCTCGGTGAAACGGCAGATGCGGAGTGAAGTTCC
HJ-3-tttc	GGAACTTCACTCCGCATCTGCCG**TTTC**TGGCTGTGGCGTGTTTCTGGTGG
HJ-4	CCACCAGAAACACGCCACAGCCAGAAAGCCGATTGCGAGGCCGTCCTACC
D-5	GGTAGGACGGCCTCGCAATCGGCTTTCTGGCTGTGGCGTGTTTCTGGTGG
J221	CAATCGGCTTTGACCTTTGGTCAATCGGCAGAT
J222	ATCTGCCGATTCTGGTTTCCAGAAAGCCGATTG
J221m	CAATCGGCATTCACCTTTGGTGATTCGGCAGAT
J222m	ATCTGCCGATTCTGGTTTCCAGATTGCCGATTG

=, phosphate modification; X, thiol-modified guanine. Cognate sequences are shown in bold.

Supplementary Figures

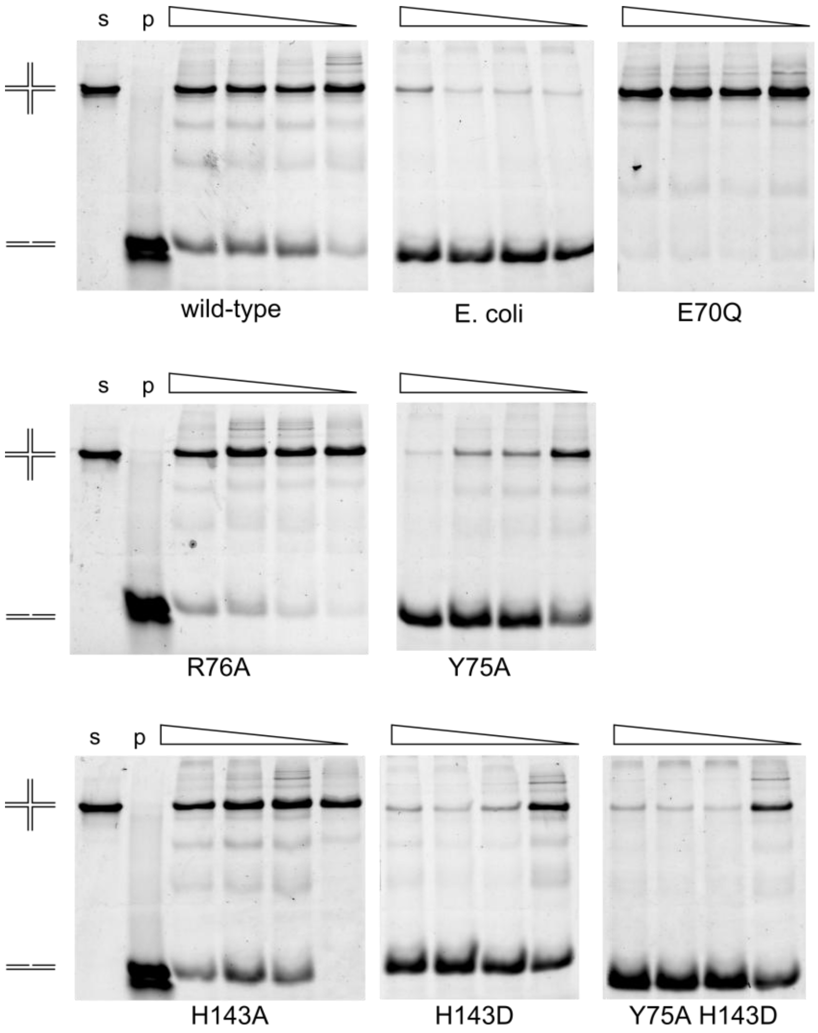

Supplementary Figure 1. Activity of Tt-RuvC and its variants. Holliday junction resolving activity was tested on substrates with fluorescently labeled non-cleaved strands, and the reaction products (nicked double-stranded DNA) were resolved on native PAGE. The protein was mixed with the substrate at 20:1 to 1:1 ratios indicated by a triangle. The protein tested in each experiment is given under the panels. Lanes denoted “s” and “p” contained the substrate and expected product of the reaction. The bands visible between the substrate and the product are assumed to correspond to the products of single HJ cleavage (nicking) (21).

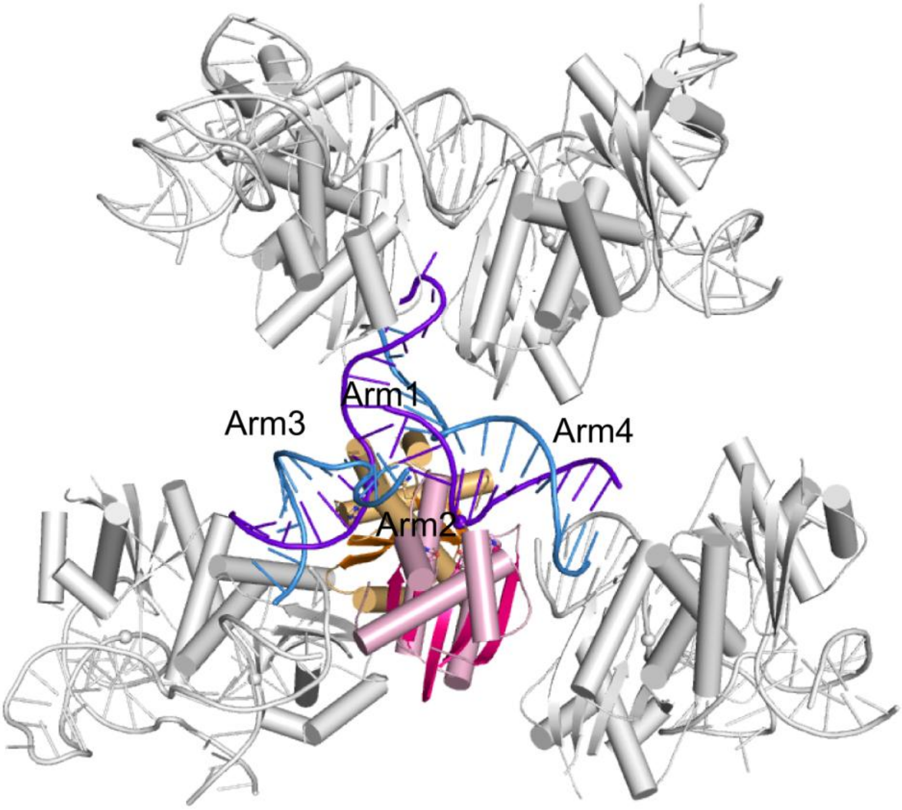

Supplementary Figure 2. Crystal packing. The protein dimer is shown in pink and orange, and the DNA is shown in purple and blue. Symmetry-related molecules are shown in gray. Arms 1 and 2 contained the three-thymine loops in the ends.

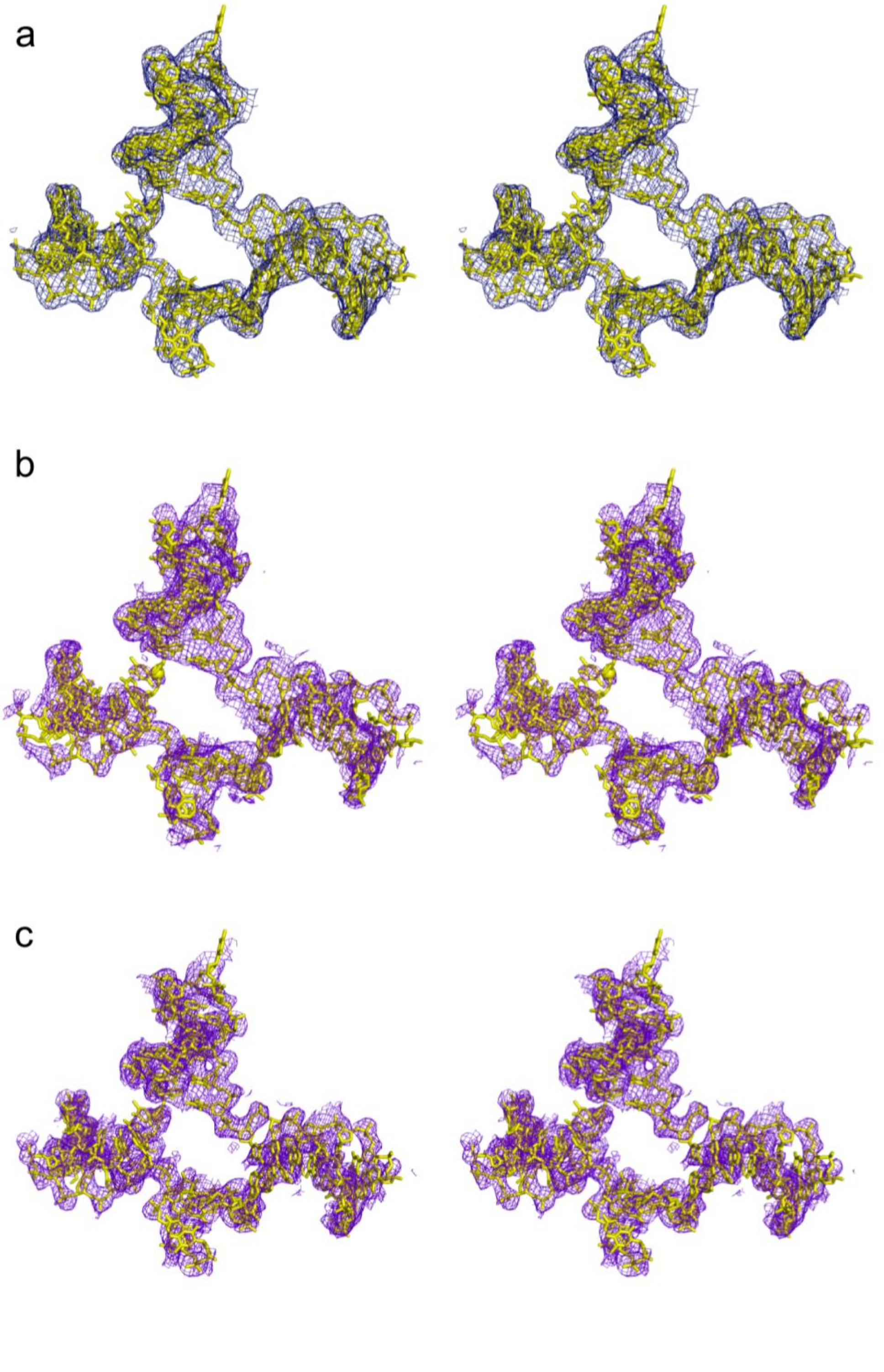

Supplementary Figure 3. **Electron density maps**. (a) The DNA structure (yellow) with overlaid 2Fo-Fc electron density map after refinement contoured at 1.5 RMS. (b) Simulated annealing omit map calculated in CNS (Crystallography and NMR System). One percent of

the model was omitted at each step, and torsion-angle molecular dynamics was performed at 1500 K. Contour level: 1 σ. (c) Fo-Fc omit maps. A bias-free model was prepared by rigid body superposition on the Tt-RuvC-DNA complex of the unliganded Tt-RuvC structure and fragments of ideal B-form DNA. Three to four nucleotides were removed at one time from the model, and it was refined in Phenix. The Fo-Fc maps from each such refinement are overlaid on the fragment of the DNA that was omitted. $\sigma = 1.5$.

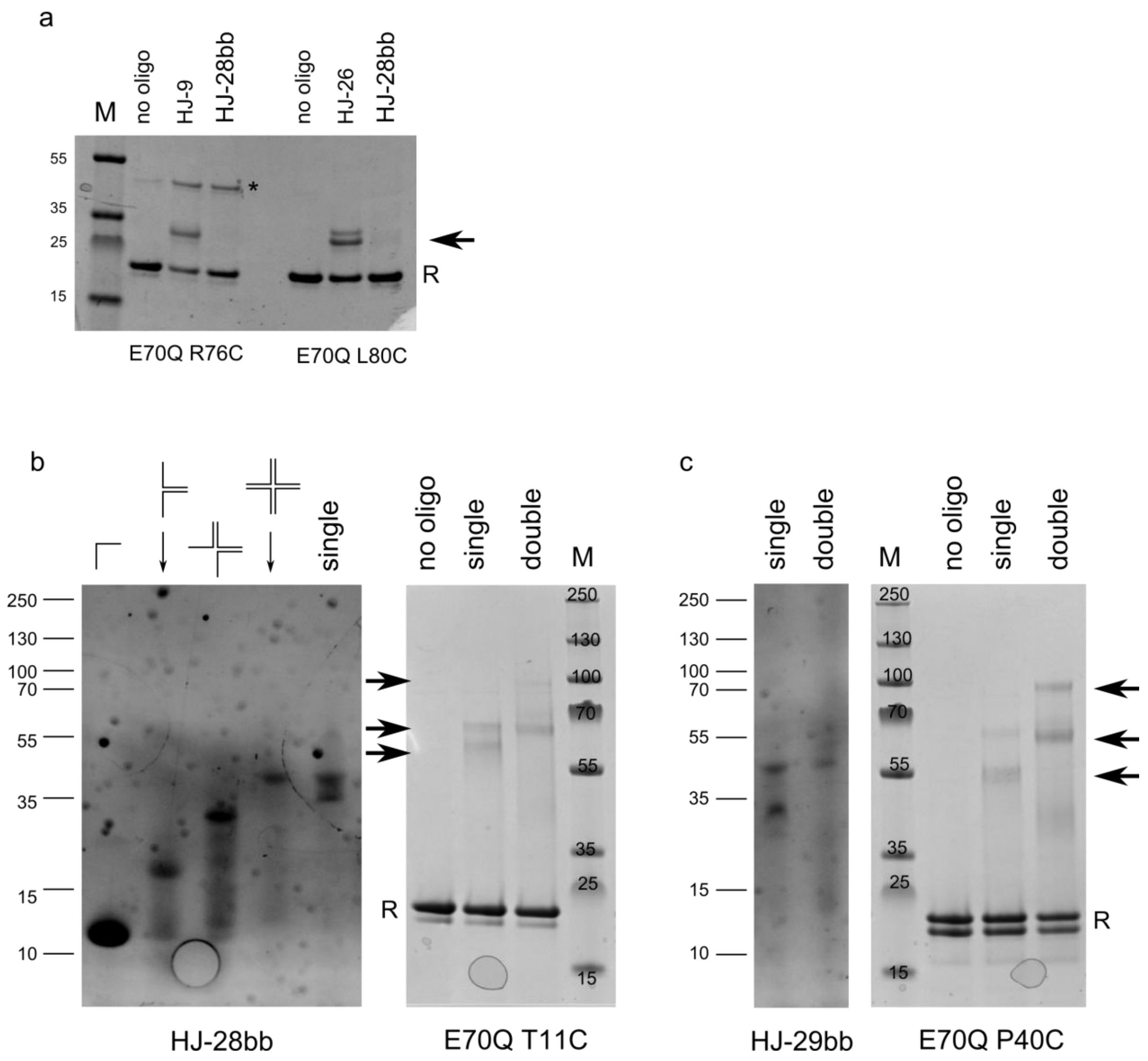

Supplementary Figure 4. Chemical cross-linking: control reactions. (a) Oligonucleotides indicated on top of the gel lanes were mixed with the protein variant given in the bottom. The cross-linking product is indicated with an arrow, and the covalent RuvC dimer is indicated with an asterisk. (b) Stability of the backbone-modified HJ (HJ-28bb) and its cross-linking with Tt-RuvC E70Q T11C. Left panel: DNAs that resulted from the stepwise addition of HJ strands with double modification (schematically shown on top of the gel) or a HJ with a single modification (designated "single") were separated on an SDS Bis-Tris gel (NuPAGE) and stained with SYBR Gold Nucleic Acid Gel Stain (Invitrogen). Right panel: Cross-linking reaction between Tt-RuvC E70Q T11C and HJ-28bb with a single ("single") modification or two symmetrical modifications ("double"). (c) Stability of the backbone-modified HJ29bb

and its cross-linking with Tt-RuvC E70Q P40C. Left panel: Single modification and double modification on SDS Bis-Tris gel stained with SYBR Gold Nucleic Acid Gel Stain (Invitrogen). Right panel: Cross-linking reaction between Tt-RuvC E70Q P40C and HJ-28bb with a single (“single”) modification or two symmetrical modifications (“double”). Protein molecular weight markers are in lanes denoted “M” and labeled. The RuvC band is marked with “R,” and cross-linking products are indicated with arrows.
